# The Performance of Continuous Glucose Monitoring During the Intraoperative Period: A Scoping Review

**DOI:** 10.3390/jcm13206169

**Published:** 2024-10-16

**Authors:** Hyun Ah Lim, Minjoo Kim, Na Jin Kim, Jaewon Huh, Jin-Oh Jeong, Wonjung Hwang, Hoon Choi

**Affiliations:** 1 Department of Thoracic and Cardiovascular Surgery, Seoul St. Mary’s Hospital, College of Medicine, The Catholic University of Korea, Seoul 06591, Republic of Korea; limzziro@catholic.ac.kr; 2 Department of Anesthesiology and Pain Medicine, Seoul St. Mary’s Hospital, College of Medicine, The Catholic University of Korea, Seoul 06591, Republic of Korea; minju1025@catholic.ac.kr (M.K.); ether@catholic.ac.kr (J.H.); 3 Medical Library, The Catholic University of Korea, Seoul 06591, Republic of Korea; kimnj@catholic.ac.kr; 4 Wake Forest Institute for Regenerative Medicine (WFIRM), Wake Forest School of Medicine, Winson-Salem, NC 27157, USA; jijeong@wakehealth.edu

**Keywords:** continuous glucose monitoring, intraoperative glycemic control, perioperative dysglycemia, intraoperative monitoring, perioperative care

## Abstract

**Introduction:** Perioperative dysglycemia is associated with negative surgical outcomes, including increased risk of infections and longer hospital stays. Continuous glucose monitoring (CGM) provides real-time glucose data, potentially improving glycemic control during surgery. However, the performance of CGM in the intraoperative environment has not been well established. This scoping review aimed to evaluate the performance of CGM systems during the intraoperative period, focusing on their technical reliability, accuracy, adverse device effects, and efficacy. **Inclusion criteria:** Studies that assessed intraoperative CGM performance, focusing on technical reliability, accuracy, adverse effects, or efficacy, were included. No restrictions were placed on the study design, surgical type, participant demographics, or publication date. **Methods:** A comprehensive literature search was performed using PubMed, EMBASE, and the Cochrane Library, covering publications up to 12 June 2024. Two independent reviewers screened and selected the studies for inclusion based on predefined eligibility criteria. Data extraction focused on the study characteristics, CGM performance, and outcomes. **Results:** Twenty-two studies were included, the majority of which were prospective cohort studies. CGM systems demonstrated a high technical reliability, with sensor survival rates above 80%. However, the accuracy varied, with some studies reporting mean or median absolute relative differences of over 15%. The adverse effects were minimal and mainly involved minor skin irritation. One randomized trial found no significant difference between CGM and point-of-care glucose monitoring for glycemic control. **Conclusions:** Although CGM has the potential to improve intraoperative glycemic management, its accuracy remains inconsistent. Future research should explore newer CGM technologies and assess their impact on surgical outcomes.

## 1. Introduction

Surgical procedures induce substantial physiological stress, which can lead to dysglycemia in both diabetic and non-diabetic individuals [1,2]. The incidence of perioperative dysglycemia is notably high, affecting 35–50% of patients undergoing non-cardiac surgeries and as many as 60–80% of those undergoing cardiac surgeries [3,4,5]. A range of perioperative factors, including hormonal fluctuations and metabolic disruptions, contribute to this elevated risk [1]. The surgical stress response elicits the release of neuroendocrine hormones, including epinephrine, glucagon, cortisol, and inflammatory cytokines such as interleukin-6 and tumor necrosis factor-alpha [6]. Collectively, these changes result in insulin resistance, reduced glucose utilization, impaired insulin secretion, increased lipolysis, and protein catabolism, all of which can exacerbate hyperglycemia [1]. Certain interventions, such as the administration of steroids for postoperative nausea and vomiting, can further increase glucose levels [7], whereas volatile anesthetics can suppress insulin secretion [8]. In cardiac surgeries, additional variables such as cardiopulmonary bypass, heparin administration, and hypothermia also contribute to dysglycemia [2]. Moreover, perioperative fasting and insulin therapy can increase the risk of hypoglycemia, which is particularly concerning because sedation and anesthesia obscure the typical signs of hypoglycemia [9,10].

The association between perioperative dysglycemia and adverse surgical outcomes, including an increased risk of postoperative infections, prolonged hospital stays, and elevated mortality rates, is well-established in both cardiac and non-cardiac surgeries [11,12]. In particular, research indicates that approximately 30–40% of patients with perioperative dysglycemia develop postoperative complications in cardiac surgery. Hyperglycemia, especially when glucose levels exceed 200 mg/dL, is associated with an increased risk of mortality, myocardial infarction, and other severe complications [12,13]. Furthermore, glycemic variability and elevated mean glucose levels are linked to longer hospital stays and a higher incidence of postoperative complications in both diabetic and non-diabetic patients undergoing non-cardiac surgery [14]. Hyperglycemia has been demonstrated to impair immune function through the disruption of chemotaxis and phagocytosis, increased adhesion molecule expression, impairment of complement pathways, and reduced nitric oxide production [15]. This cascade of dysfunctions results in increased inflammation, susceptibility to infection, and multiorgan complications [16]. Conversely, severe hypoglycemia, even when transient, can lead to neuronal damage, particularly in the cortex, hippocampus, and basal ganglia, resulting in significant cognitive impairment [17]. The risk of hypoglycemia is emphasized by findings from the NICE-SUGAR trial, which associated intensive glucose control with an elevated risk of hypoglycemia-related mortality [18].

Considering these risks, effective perioperative glucose monitoring is essential [19]. Conventional methodologies, including central laboratory tests, blood gas analyzers, and point-of-care (POC) glucometers, exhibit limitations, particularly regarding real-time monitoring, as they provide intermittent glucose measurements that cannot reliably detect asymptomatic or nocturnal hypoglycemia [20]. This underscores the necessity for continuous glucose monitoring (CGM) technologies that offer real-time data, enhance the identification of glycemic trends, and facilitate timely intervention [21].

Over the past two decades, advancements in diabetes technology have resulted in the development of CGM devices that can be either subcutaneous or intravascular [22]. Subcutaneous devices, which are the most widely used, continuously measure interstitial glucose levels and transmit data to a receiver in real-time [23]. Conversely, intravascular CGMs are more invasive, but provide superior accuracy [24]. In contrast to traditional methods, which provide only intermittent readings, CGM offers continuous glucose readings, facilitating the rapid detection of glycemic fluctuations, reducing the incidence of hypoglycemia and hyperglycemia episodes, and eliminating the need for frequent finger sticks [19,21]. While laboratory-based glucose analysis remains important, particularly in the intensive care unit (ICU) or recovery settings, CGM provides continuous real-time data, enabling early detection of glycemic fluctuations during surgery and the immediate postoperative period, when laboratory results may be delayed, or glucose levels may change rapidly. Continuous monitoring by CGM allows for the detection of asymptomatic hypoglycemia or hyperglycemia events that might go unnoticed with intermittent monitoring and provides a clearer picture of glycemic variability. The capacity of CGM to detect asymptomatic events and glycemic variability enhances overall glucose management and patient outcomes [23].

While CGM was initially designed for outpatient use [23], its applications have expanded to inpatient settings, particularly during the COVID-19 pandemic [25,26]. Research indicates that CGM in non-ICU hospital settings can mitigate both hypo- and hyperglycemia [27,28]. Although concerns persist regarding its accuracy in critically ill patients, specifically those experiencing impaired tissue perfusion, hypotension, and hypoxia, recent studies have demonstrated its feasibility in the ICU setting [29,30]. Notably, CGM accuracy has been validated even in patients suffering from shock or receiving vasopressors [29,31].

Despite these advances, the intraoperative environment presents significant challenges for the utilization of CGM. Fluid shifts, tissue edema, and vasopressor administration during surgical procedures can result in interstitial hypoperfusion, potentially compromising CGM accuracy [1]. Vasopressors, while essential for maintaining systemic blood pressure, cause vasoconstriction that can reduce microcirculatory blood flow to tissues [32]. This diminished perfusion in the interstitial space can impair glucose transport to the CGM sensor, thus affecting the accuracy of glucose readings. Furthermore, interference from surgical devices, such as diathermy and radiation equipment, may lead to malfunction or signal loss [33]. Nonetheless, the potential for CGM application during the intraoperative period remains substantial, particularly during critical surgical phases when glucose regulation is most volatile [10].

Recently, studies examining the use of CGM during the intraoperative period have accumulated; however, no comprehensive review has been conducted to evaluate the available evidence. To address this gap, this scoping review aims to evaluate the current literature on intraoperative CGM use, map out its performance, and identify gaps in knowledge. By synthesizing the existing evidence, this review aims to provide useful insights into the use of CGM during the intraoperative period and to highlight potential areas for future research.

## 2. Materials and Methods

### 2.1. Study Design

The rationale for choosing a scoping review was to accommodate the anticipated heterogeneity in study designs and reported outcomes within this research domain. This review adheres to the guidelines outlined in the Preferred Reporting Items for Systematic Reviews and Meta-Analyses extension for Scoping Reviews (PRISMA-ScR; Supplementary Materials Table S1) [34]. The protocol for this review is available upon reasonable request from the corresponding author.

### 2.2. Eligibility Criteria

This review included studies that evaluated the performance of CGM systems during the intraoperative period. Eligible studies were required to assess at least one of the following CGM performance metrics: technical reliability, accuracy, adverse device effects, and efficacy. There were no restrictions on the publication date, language, participant age, underlying medical conditions, or type of surgery. Studies that focused exclusively on the preoperative or postoperative periods without assessing intraoperative CGM performance were excluded. In addition, studies that did not provide sufficient data on CGM performance were excluded. Articles reporting hybrid closed-loop systems were included only if they provided performance data that were specific to the CGM component. Studies involving the STG closed-loop system by Nikkiso (Tokyo, Japan) were excluded because this device is an artificial pancreas system available only in Japan [35]. Animal studies, abstracts, editorials, book chapters, dissertation works, and reviews were excluded.

### 2.3. Source of Evidence and Search Strategy

A comprehensive literature search was conducted across PubMed, EMBASE, and the Cochrane Library from their inception to 12 June 2024. The search strategy for each database was developed by an experienced librarian (N.J.K.) and refined through consultation with the research team. The search strategy for PubMed is presented in Table 1, and the full search strategies are available in the Supplementary Materials (Table S2). Manual reference list searches were also performed to identify additional relevant studies.

All identified references were imported into EndNote (version 21.4; Clarivate Analytics, Philadelphia, PA, USA) and duplicate entries were removed. The results were subsequently imported into Rayyan (https://www.rayyan.ai/ (accessed on 12 June 2024)) for blind screening [36]. Two independent reviewers (H.A.L. and H.C.) conducted title and abstract screening and full-text reviews were performed for potentially eligible studies. Discrepancies were resolved through discussion or, when necessary, by consultation with a third reviewer (M.K.).

### 2.4. Data Extraction

A standardized data extraction form was developed and pilot-tested on four articles. Two reviewers (H.A.L. and H.C.) independently extracted the data and any discrepancies were resolved through consultation with a third reviewer (M.K.). The extracted data included the following: study characteristics, design, sample size, type of surgery, CGM characteristics, and performance outcomes. The CGM performance was evaluated in accordance with the Performance Metrics for Continuous Interstitial Glucose Monitoring outlined by the Clinical and Laboratory Standards Institute (CLSI Guideline POCT05; Table 2) [37]. For studies reporting perioperative data, only intraoperative data were extracted, when feasible.

### 2.5. Data Synthesis

The extracted data were qualitatively synthesized to map the current state of evidence regarding CGM use during the intraoperative period. Key themes and findings were summarized narratively, and the results were categorized by CGM performance metrics including technical reliability, accuracy, adverse effects, and efficacy.

## 3. Results

### 3.1. Selection of Sources of Evidence

**Figure 1:**
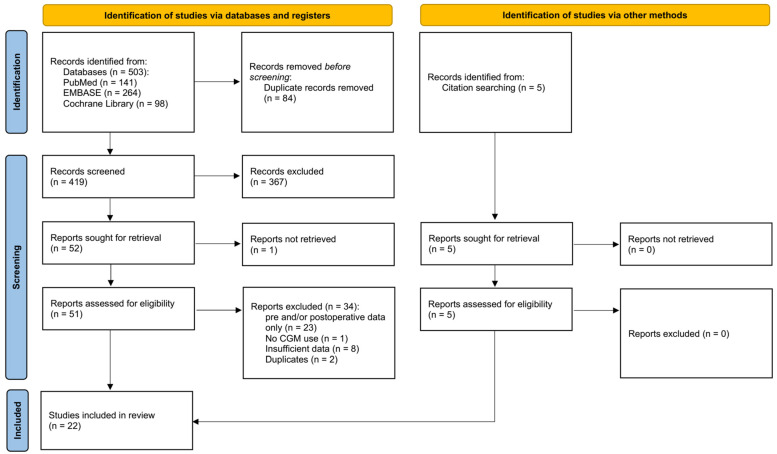
PRISMA flow diagram of included and excluded citations in this study.

A total of 503 articles were identified through electronic database search. After the removal of duplicates, 419 articles remained. Screening of titles and abstracts resulted in the exclusion of 367 articles, leaving 52 full-text articles to be assessed for eligibility. Of these, 35 articles were excluded for the following reasons: 23 focused exclusively on preoperative or postoperative data, one did not utilize CGM technology, eight did not provide sufficient performance data, one could not be retrieved despite multiple attempts to contact the authors, and two were duplicates. Five additional eligible studies were identified through manual reference search. Ultimately, 22 studies were included in this review (Figure 1).

### 3.2. Study Characteristics

The characteristics of the included studies are shown in Table 3. The studies were conducted between 2005 and 2023, representing diverse geographical distributions across North America, Europe, and Asia. The predominant study design (*n* = 16) was a prospective cohort study, while two were randomized controlled trials (RCTs) and four were case reports. The sample sizes varied, with the majority of studies focusing on adult populations (*n* = 17), although three studies included children and two studies involved neonates. The most prevalent surgical categories were abdominal (*n* = 10) and cardiac (*n* = 14) surgeries, reflecting the heterogeneous range of surgical procedures evaluated.

### 3.3. CGM Characteristics

The studies employed various CGM systems, as shown in Table 4. Most studies (*n* = 18) exclusively used subcutaneous CGM devices [38,40,41,42,43,44,45,46,48,49,50,53,54,55,56,57,58,59], three studies used intravascular CGM systems [39,47,52], and one study used both types [51]. Medtronic CGM devices were the most frequently used (*n* = 10), including Enlite [50,53,55,56], Guardian REAL-Time [46,48,54], and the CGMS system Gold [38,44,58]. Other CGM systems included Dexcom G6 (*n* = 7) [40,41,42,43,45,49,57], Abbott Freestyle Libre (*n* = 3) [49,51,59], and A. Menarini Diagnostics GlucoDay (*n* = 1) [58]. Two intravascular CGM systems, the Eirus Microdialysis system (*n* = 3) [39,51,52] and the Edwards Lifescience GlucoClear (*n* = 1) [47], were also employed. While the majority of studies provided data covering both intraoperative and postoperative periods, five studies focused exclusively on the intraoperative phase [42,43,44,56,58].

### 3.4. Technical Reliability

The technical reliability outcomes were reported in 18 studies (Table 5). Sensor survival, defined as the ability of a CGM sensor to function for the intended duration of use, was assessed in 13 studies. In 12 of these studies, sensor survival rates exceeded 80%, indicating high reliability [38,42,43,48,49,50,52,53,54,55,57,58]. Two studies reported lower survival rates of approximately 50%; however, one of these studies was a case report involving only two patients undergoing cardiac surgery [41], and the other study used an older CGM system (A. Menarini Diagnostics GlucoDay) [58]. Data availability, defined as the proportion of successful glucose measurements without interruption, was reported in five studies. Three of these studies reported data availability rates above 95% [38,40,57], whereas one study reported lower availability (58.7% and 72.9% for abdominal and thigh sensor placement, respectively) [54]. This study extended the CGM data collection into the postoperative period, which may have influenced the lower availability rates.

Several studies reported additional technical challenges. For example, interruptions occurred in 22 of 24 patients (91.7%) using the Eirus Microdialysis system, with a mean data gap duration of 13 ± 19 min. One notable instance involved a data gap of 141 min due to a calibration complication. These interruptions were primarily attributed to calibration issues, catheter-related complications, and technical malfunctions. Despite these interruptions, the system performed adequately during the intraoperative period [51]. In the same study, technical failure due to sensor detachment was reported in a patient using the Abbott Freestyle Libre [51]. In another study, interference from electrocautery during surgery triggered false alarms in 50% of the participants [46].

### 3.5. Accuracy

Accuracy was assessed in 18 studies using various methods, including comparisons with arterial, venous, and capillary blood glucose samples (Table 6). The most commonly reported accuracy metric was the mean or median absolute relative difference (MARD), which quantifies the percentage difference between the CGM readings and reference glucose measurements. A lower MARD value indicates a better CGM accuracy. Eight studies reported MARD values below 15%, which is generally considered acceptable for clinical use [39,42,45,47,51,52,57,58], whereas five studies reported MARD values exceeding 15%, indicating potential accuracy issues during intraoperative use [43,44,46,51,54].

The International Organization for Standardization (ISO) criteria, which set regulatory standards for glucose monitoring devices, were used to evaluate the CGM accuracy in four studies [45,51,52,57]. According to the 2003 ISO guidelines (ISO15197:2003) [60], for devices to meet approval, 95% of test glucose values must be within ± 20% of the reference value if the glucose level is greater than 75 mg/dL, or within ± 15 mg/dL if the reference value is lower [60]. The 2013 update (ISO15197:2013) tightened the criteria, requiring 95% of values to fall within ± 15% for glucose levels greater than 99 mg/dL and within ± 15 mg/dL for values lower than 99 mg/dL [61]. In our review, only one study that compared the Eirus Microdialysis system with arterial blood gas analysis met the ISO criteria, with over 95% of CGM readings within the acceptable range [52].

The Bland–Altman method, used in 10 studies, evaluates the agreement between CGM and reference blood glucose measurements by calculating the mean bias (the average difference between CGM and reference values) and the limits of agreement (mean bias ± 1.96 standard deviations) [39,41,47,49,51,52,54,55,57,59]. The mean bias represents the systematic difference between the two methods, with absolute mean biases ranging from less than 20 mg/dL in nine studies [41,47,49,51,52,54,55,57,59] to over 20 mg/dL in six studies [41,49,51,54,55,59]. The limits of agreement, which indicate the variability in CGM accuracy, varied significantly across studies. Although the mean bias was generally low across studies, many studies reported wide limits of agreement, suggesting variability in CGM accuracy during surgery.

Error grid analysis was used in 11 studies to evaluate the clinical accuracy of the CGM [38,39,43,45,46,51,52,54,55,57,58]. This method categorizes paired glucose measurements into different zones based on their potential to affect clinical decision making [62]. Zone A includes clinically accurate values, meaning that they do not lead to inappropriate treatment decisions. Zone B includes values that are acceptable but might prompt unnecessary, although harmless, treatment. Zones C, D, and E represent glucose measurements that could lead to inappropriate clinical decisions and potentially cause harm. In nine studies, more than 95% of CGM readings fell within zones A and B, indicating that the majority of CGM measurements were clinically acceptable for intraoperative use [38,39,45,46,51,52,55,57,58].

Finally, the association between CGM and reference measurements was analyzed in eight studies using correlation and regression methods, including Pearson’s correlation and intraclass correlation [38,39,41,46,48,49,54,59]. All these studies found statistically significant correlations, further supporting the reliability of CGM devices, although the strength of these associations varied across studies.

### 3.6. Adverse Device Effects

Ten studies reported adverse device effects (Table 7). Nine studies reported no adverse device effects [38,39,40,43,46,48,52,54,59]. One study documented minor adverse events, including self-limited bleeding at the sensor insertion site, mild pruritus, and skin irritation [57]. Other studies explicitly reported no incidence of local infection, thrombophlebitis, or sensor dislodgment [46,54,59].

### 3.7. Efficacy

Only one study assessed the efficacy of CGM during the intraoperative period. This RCT involved patients with type 2 diabetes undergoing major abdominal or cardiothoracic surgery, comparing glycemic management guided by intravascular CGM (CGM-ON) with standard POC glucometer monitoring (CGM-OFF) [47]. The study found no statistically significant differences between the two groups in terms of glycemic control.

Postoperative median glucose levels were comparable between the groups, with the CGM-ON group having a median glucose level of 153 mg/dL (interquartile range [IQR]: 122–185 mg/dL) compared with 167 mg/dL (IQR: 149–189 mg/dL) in the CGM-OFF group (*p* = 0.50). Additionally, the proportion of glucose readings within the target range was higher in the CGM-ON group, with 93% (IQR: 71–100%) of the measurements falling within the target range, compared to 72% (IQR: 46–100%) in the CGM-OFF group, although this difference was not statistically significant (*p* = 0.09). Similarly, the proportion of POC glucometer measurements in the target range was slightly higher in the CGM-ON group (75% [IQR: 46–100%]) than in the CGM-OFF group (67% [IQR: 27–100%]), but this difference was not statistically significant (*p* = 0.56). While these results suggest a trend toward better glycemic control in the CGM group, the lack of statistical significance indicates that further research is needed to determine the clinical efficacy of CGM-guided glycemic management during surgery.

## 4. Discussion

### 4.1. Summary of Main Findings

This scoping review evaluated the performance of CGM systems during the intraoperative period with a focus on technical reliability, accuracy, adverse device effects, and efficacy. Across the included studies, CGM demonstrated a generally high technical reliability, with sensor survival rates exceeding 80% and data availability rates above 95% in most cases. However, certain challenges were observed, particularly in older CGM models and during surgeries involving complex physiological changes, such as cardiac surgery. The results for accuracy were mixed, with several studies reporting MARD values below 15%; however, the accuracy often fluctuated depending on the surgical context and device model. Adverse device effects were rare, and the limited efficacy data from one RCT found no statistically significant differences between CGM and standard POC glucometer monitoring, although there was a trend toward improved glycemic control with CGM.

### 4.2. Comparison with Existing Literature

Our review provides new insights into the intraoperative use of CGM, a topic that has been relatively underexplored compared to its use in outpatient and inpatient settings. CGM is widely recognized in outpatient settings because it improves glycemic control, reduces the frequency of hypoglycemia and hyperglycemia, and enhances patient satisfaction by minimizing the need for finger stick blood glucose tests [63,64,65,66]. These benefits, along with the need for continuous glucose monitoring in complex perioperative environments where rapid physiological changes occur, have prompted efforts to integrate CGM into perioperative care. In particular, the potential to detect and manage asymptomatic glucose fluctuations, improve glycemic variability control, and provide real-time glucose data during critical periods has driven this interest. However, the unique challenges of the intraoperative environment, such as rapid physiological changes, fluid shifts, and technical interferences, complicate CGM implementation [19,67].

The findings from our review align with existing studies in critical care settings, such as the ICU, where CGM performance is affected by factors such as impaired tissue perfusion, vasopressor use, and rapid physiological changes [29,68]. Similarly, in the intraoperative period, fluid shifts and anesthesia can cause discrepancies between the interstitial glucose levels measured by CGM and the actual blood glucose levels [69]. Despite these challenges, many of the reviewed CGM systems have maintained a reasonable degree of reliability and data availability during surgery, although their accuracy could be compromised under certain conditions.

While some of our findings confirm earlier conclusions about CGM accuracy issues in critical care settings, our review also sheds light on intraoperative-specific challenges, such as the high rate of system interruptions and the variability of CGM accuracy depending on the type of surgery [29]. These challenges have not been comprehensively addressed in previous research, highlighting areas where further study is needed. Only one study in this review met the strict ISO criteria for accuracy, specifically the ISO 15197:2003 standard [52]. However, it is important to note that the newer ISO 15197:2013 criteria, which require stricter accuracy thresholds, were not met by most subcutaneous CGM systems [45,51,57]. This underscores the need for ongoing refinement of the CGM technology to better accommodate the unique physiological demands of the intraoperative environment.

Another critical issue that remains understudied is the long-term effect of CGM on surgical outcomes. Although CGM has been shown to improve glycemic control in outpatient settings, its efficacy in improving postoperative outcomes, such as reducing complications and mortality, is yet to be established. Only one study in our review assessed CGM efficacy and found no significant differences in postoperative glucose control between CGM and POC glucometer monitoring [47]. Further research is required to determine the impact of CGM on perioperative morbidity and mortality.

### 4.3. Clinical Implications

The perioperative period represents a critical window for glycemic control, particularly in patients undergoing major surgeries such as abdominal and cardiac procedures [1]. Dysglycemia during this period has been associated with an increased risk of postoperative complications, including infections, delayed wound healing, and prolonged hospital stay [1]. Given these risks, CGM has the potential to transform intraoperative glycemic management by providing real-time glucose data that allow for more timely interventions.

One of the key advantages of CGM in the OR is its ability to continuously monitor glucose levels, offering minute-to-minute insights into glucose trends [22]. Traditional methods, such as POC glucometers or central laboratory tests, only provide intermittent glucose readings, which can miss rapid fluctuations that occur due to surgical stress or anesthesia [19]. This limitation is especially significant in surgeries in which the physiological stress response can cause sudden shifts in glucose levels [69]. In contrast, CGM can alert clinicians to impending hypoglycemia or hyperglycemia, enabling more proactive glycemic management [70].

Reducing glycemic variability, which refers to the degree of fluctuation in glucose levels, may be particularly important in surgeries requiring tight glucose control, such as cardiac surgeries [71]. Evidence suggests that glycemic variability, independent of mean glucose levels, contributes to an increased risk of postoperative complications, including infections and mortality [72,73,74]. By continuously monitoring glucose levels, CGM may help mitigate fluctuations and maintain a more stable glucose profile during surgery, potentially improving surgical outcomes.

However, the limitations of CGM in the intraoperative environment cannot be overlooked. As demonstrated in our review, the CGM accuracy is not always consistent, particularly in cases where rapid physiological changes occur. For instance, inaccuracies in CGM data during surgery can lead to inappropriate insulin dosing, resulting in iatrogenic hypoglycemia [69]. Given these limitations, CGM should be considered an adjunct to traditional glucose monitoring methods rather than a replacement. While CGM provides the advantage of continuous real-time monitoring, traditional methods, such as POC glucometer, offer a more reliable reference, particularly during critical moments when accuracy is paramount. Clinicians must continue to validate CGM readings with reference measurements such as POC tests, particularly when discrepancies arise. Ultimately, the integration of CGM into intraoperative care protocols will require careful consideration of its accuracy, reliability, and potential impact on clinical decision making.

### 4.4. Technical Limitations of CGM in the Intraoperative Period

The use of CGM during surgery introduces unique technical challenges that affect its accuracy and reliability. One of the most significant issues is the effect of intraoperative physiological changes such as fluid shifts, blood loss, and tissue hypoperfusion on CGM performance [67]. These changes can lead to discrepancies between CGM interstitial glucose readings and actual blood glucose levels, particularly in patients undergoing major surgeries such as cardiac or abdominal procedures.

Anesthesia also plays a role in complicating CGM accuracy. Certain anesthetic agents, such as volatile anesthetics, can reduce insulin secretion and contribute to intraoperative hyperglycemia [8]. Additionally, anesthetized patients are unable to exhibit typical hypoglycemic symptoms, making it challenging to detect low glucose levels without continuous monitoring [75]. In such cases, sole reliance on CGM could lead to missed episodes of hypoglycemia if the device fails to capture rapid declines in glucose levels [70]. Additionally, CGM devices have been shown to be less accurate in detecting extreme glucose levels, including severe hyperglycemia and hypoglycemia [76].

Mechanical and environmental factors in the operating room further complicate the use of CGM. Devices such as electrocautery and radiation machines can interfere with CGM sensor functionality by generating electromagnetic interference which can disrupt the sensor’s ability to transmit accurate glucose data [77,78]. This interference may result in signal loss, inaccurate readings, or false alarms, potentially compromising the reliability of CGM during surgical procedures [79]. These disruptions highlight the need for careful monitoring and validation of CGM readings in environments with high electromagnetic activity. Similarly, surgical drapes and patient positioning may dislodge sensors, leading to data loss or sensor failure. In cardiac surgeries, particularly those involving cardiopulmonary bypass, additional factors, such as hemodilution, hypothermia, and altered tissue perfusion, can further affect CGM accuracy [2].

Addressing these technical limitations is critical for improving CGM reliability in the intraoperative setting [20]. Future research should focus on developing more robust sensors that are resistant to environmental interference, and better algorithms that account for the rapid physiological changes observed during surgery.

### 4.5. Strengths and Limitations of the Review

This scoping review has several strengths, including a comprehensive search strategy that covers multiple databases and a wide variety of studies from different geographical locations and surgical contexts. This review also considered both subcutaneous and intravascular CGM systems, providing a broad assessment of CGM performance during surgery.

However, this review has some limitations which must be noted. The heterogeneity of the included studies, particularly in terms of study design, sample size, and CGM systems used, limits the ability to draw definitive conclusions regarding the overall performance of CGM in intraoperative settings. Furthermore, the majority of studies focused on subcutaneous CGM devices, whereas relatively few evaluated intravascular CGMs, which may offer better accuracy in the surgical environment.

Another limitation was the lack of large RCTs assessing the clinical efficacy of CGM during surgery. While one trial compared CGM-guided glucose management with POC glucometer monitoring, the results were inconclusive [47]. More robust trials are needed to determine whether CGM can improve the surgical outcomes.

### 4.6. Future Research Directions

Given the current limitations of CGM in intraoperative settings, future research should focus on several key areas. First, larger RCTs are needed to evaluate the clinical efficacy of CGM in improving perioperative outcomes such as reducing dysglycemia, postoperative infections, and mortality [80]. These studies should assess CGM performance across various surgical populations, including high-risk patients undergoing major surgery.

Second, future research should prioritize the assessment of newer CGM devices, such as Medtronic Guardian 4, Abbott Freestyle Libre 3.0, and Dexcom G7, which have demonstrated improved accuracy and patient comfort in outpatient settings [81,82,83]. However, their performance in the intraoperative environment remains largely unexplored, and rigorous studies are required to evaluate their accuracy and reliability during surgery.

Additionally, future studies should use standardized accuracy metrics, such as ISO criteria [61], CLSI Guideline POCT05 [37], and FDA iCGM approval standards [84], to ensure that CGM systems meet the necessary benchmarks for clinical use. Further investigation into the role of glycemic variability during surgery is also needed, as stabilizing glycemic fluctuations may prove essential for improving patient outcomes [74].

Finally, future research should continue to examine the safety and tolerability of CGM devices in surgical patients. Ensuring that these devices are not only accurate but also safe and practical for intraoperative use, especially in high-risk populations such as patients with diabetes or those undergoing complex procedures, will be crucial for their integration into routine perioperative care [67].

## 5. Conclusions

This review highlights that, although CGM provides continuous real-time data, significant challenges currently limit its effectiveness in improving intraoperative glycemic management. Inconsistencies in CGM accuracy, particularly during periods of rapid physiological changes, limit its reliability for critical intraoperative decisions. Many studies have demonstrated variability in CGM performance, with some reporting MARD values above 15%, indicating that current CGM technology may not yet be sufficiently accurate for routine intraoperative use. Furthermore, CGM sensors are susceptible to interference from intraoperative factors such as fluid shifts, vasopressors, and surgical equipment, which can lead to signal loss or inaccurate readings.

Given these limitations, CGM should currently be considered an adjunct to traditional glucose monitoring methods rather than a replacement. However, with advancements in sensor technology, improvements in accuracy, and more robust clinical trials to establish efficacy, CGM could become a valuable tool in intraoperative glycemic management in the future. Further research is needed to overcome the current challenges and to better understand the role of CGM in improving perioperative outcomes.

## Figures and Tables

**Table 1:** Search strategy for PubMed.

Category	Search Terms
Population	
1	“Surgical Procedures, Operative”[Mesh]
2	“Surgical Procedures, Operative”[TW] OR “Surgical Procedures”[TW] OR “Procedures, Surgical”[TW] OR “Procedure, Surgical”[TW] OR “Surgical Procedure”[TW] OR “Operative Procedures”[TW] OR “Operative Procedure”[TW] OR “Procedure, Operative”[TW] OR “Procedures, Operative”[TW] OR “Operative Surgical Procedure”[TW] OR “Operative Surgical Procedures”[TW] OR “Procedure, Operative Surgical”[TW] OR “Procedures, Operative Surgical”[TW] OR “Surgical Procedure, Operative”[TW]
3	“General Surgery”[Mesh]
4	“General Surgery”[TW] OR “Surgery, General”[TW] OR “Surgery”[TW] OR “general surgery patient”[TW] OR “general surgery patients”[TW]
5	“surgery” [Subheading]
6	“operations”[TW] OR “invasive procedures”[TW] OR “operative therapy”[TW] OR “preoperative procedures”[TW] OR “intraoperative procedures”[TW] OR “peroperative procedures”[TW] OR “perioperative procedures”[TW]
7 Combine	1 OR 2 OR 3 OR 4 OR 5 OR 6
Concept	
8	“Continuous Glucose Monitoring”[Mesh]
9	“Continuous Glucose Monitoring”[TW] OR “Glucose Monitoring, Continuous”[TW] OR “Monitoring, Continuous Glucose”[TW] OR “Monitorings, Continuous Glucose”[TW] OR “Continuous Glucose Monitoring Device”[TW] OR “CGM Device”[TW] OR “CGM Devices”[TW] OR “Device, CGM”[TW] OR “Devices, CGM”[TW] OR “CGM”[TW]
10	(“Blood Glucose”[Mesh] OR “Blood Glucose”[TW]) AND “Continuous”[TW]
11	“Continuous Blood Glucose”
12 Combine	8 OR 9 OR 10 OR 11
Context	
13	“Perioperative Period”[Mesh]
14	“Perioperative Period”[TW] OR “Period, Perioperative”[TW] OR “Periods, Perioperative”[TW] OR “Perioperative Periods”[TW]
15	“Preoperative Period”[Mesh] OR “Preoperative Period”[TW] OR “Period, Preoperative”[TW]
16	“Intraoperative Period”[Mesh]
17	“Intraoperative Period”[TW] OR “Intraoperative Periods”[TW] OR “Period, Intraoperative”[TW] OR “Periods, Intraoperative”[TW]
18	“Postoperative Period”[Mesh]
19	“Postoperative Period”[TW] OR “Period, Postoperative”[TW] OR “Periods, Postoperative”[TW] OR “Postoperative Periods”[TW]
20 Combine	13 OR 14 OR 15 OR 16 OR 17 OR 18 OR 19
21 Combine	7 AND 12 AND 20

**Table 2:** Continuous glucose monitoring performance evaluation.

Category	Parameter	Description
Technical reliability	Sensor survival	The ability of CGM to correctly function until the end of its intended use.
	Data availability	The ability of CGM to provide the expected number of glucose measurements without interruptions.
Accuracy	Mean and/or median bias/absolute difference	Average difference between CGM value and comparator value.
	Mean and/or median absolute relative difference (MARD)	Average difference between CGM value and comparator value divided by the comparator value.
	Error grid analysis (Clarke, consensus, surveillance, and continuous glucose)	Values in Zone A and B considered clinically acceptable.
	Agreement rate	Proportion of CGM values within certain limits (e.g., ±15 mg/dL or ±15%) of comparator values.
	Limits of agreement	Indicate how closely CGM values and comparator values agree using the Bland–Altman method (mean ± 1.96 standard deviation).
	Association	Using correlation and/or regression.
Adverse device effects	Adverse device effects	Occurrence of adverse events related to or caused by CGM.
Efficacy	Average glucose level	Assessed in comparison with a comparator by at least one metric of the average glucose level.
	Time in range	Assessed in comparison with a comparator by time spent in different glucose ranges.

CGM, continuous glucose monitoring.

**Table 3:** Study characteristics of the included studies (*n* = 22).

First Author Year	Origin	Design	Population	Surgery Type	Aims
Aust 2014 [38]	Germany	Prospective cohort	Adults (*n* = 10)	Cardiac (*n* = 10)	To evaluate if subcutaneous CGM is feasible in cardiac surgery and if reliable glucose values are reported under hypothermic extracorporeal circulation.
Blixt 2013 [39]	Sweden	Prospective cohort	Adults (*n* = 10)	Abdominal (*n* = 10)	To test a central venous catheter with a microdialysis membrane in combination with an online analyzer and monitor as a principle for CGM.
Carlsson 2023 [40]	Denmark	Prospective cohort	Adults (*n* = 70)	Abdominal (*n* = 45), orthopedic (*n* = 11), vascular (*n* = 14)	To investigate the frequency and duration of hypo- and hyperglycemia, assessed by CGM during and after major surgery.
DiGiusto 2021 [41]	USA	Case report	Children (*n* = 2)	Abdominal (*n* = 2)	To assess the accuracy of CGM compared to capillary POC and arterial blood analysis in two cases where CGM was utilized as an adjunct method of perioperative glucose monitoring.
Guensch 2021 [42]	Switzerland	Case report	Adults (*n* = 2)	Cardiac (*n* = 2)	To present the first insights into the performance of the Dexcom G6 sensor during cardiac surgery with mild and deep hypothermia.
Herzig 2023 [43]	Switzerland	Prospective cohort	Adults (*n* = 16)	Cardiac (*n* = 16)	To test the accuracy of the Dexcom G6 CGM sensor in patients undergoing cardiac surgery using hypothermic extracorporeal circulation.
Kalmovich 2012 [44]	Israel	Prospective cohort	Adults (*n* = 32)	Cardiac (*n* = 32)	To examine and monitor the glycemic response in patients undergoing cardiac surgery during the perioperative period, using 24 h monitoring with a CGM and evaluating its accuracy and reliability.
Perez-Guzman 2021 [45]	USA	Prospective cohort	Adults (*n* = 15)	Cardiac (*n* = 15)	To evaluate the performance of CGM in adults without DM undergoing scheduled or urgent coronary artery bypass graft surgery.
Piper 2006 [46]	USA	Prospective cohort	Children (*n* = 20)	Cardiac (*n* = 20)	To validate a subcutaneous sensor for real-time CGM in pediatric patients during and after cardiac surgery.
Polderman 2017 [47]	The Netherlands	RCT	Adults (*n* = 36)	Major abdominal or cardiothoracic	To investigate the efficacy of perioperative CGM via peripheral intravenous sampling in patients with DM type 2 compared with standard care.
Poljakova 2013 [48]	Czech Republic	Prospective cohort	Adults (*n* = 17)	Orthopedics (*n* = 13), vascular (*n* = 4)	To explore the feasibility of subcutaneous CGM in perioperative settings.
Price 2023 [49]	USA	Prospective cohort	Adults (*n* = 76)	Abdominal (*n* = 13), cardiac (*n* = 5), otolaryngologic (*n* = 6), gynecological (*n* = 5), neuro (*n* = 14), ophthalmologic (*n* = 1), orthopedic (*n* = 6), plastic (*n* = 5), thoracic (*n* = 3), urologic (*n* = 8), vascular (*n* = 10)	To compare the performance of two CGM devices to contemporaneous capillary blood glucose sampling in patients with DM undergoing major non-cardiac surgery.
Saha 2018 [50]	United Kingdom	Case report	Neonate (*n* = 1)	Abdominal (*n* = 1)	To report on a preterm infant who uniquely underwent surgery while wearing a CGM, blinded to the clinical team.
Schierenbeck 2017 [51]	Sweden	Prospective cohort	Adults (*n* = 24)	Cardiac (*n* = 24)	To compare two different CGM systems: the FreeStyle Libre subcutaneous CGM and the Eirus intravascular microdialysis CGM in patients undergoing cardiac surgery.
Schierenbeck 2013 [52]	Sweden	Prospective cohort	Adults (*n* = 30)	Cardiac (*n* =30)	To evaluate the accuracy of intravascular microdialysis CGM in patients undergoing cardiac surgery.
Sindhvananda 2023 [53]	Thailand	RCT	Adults (*n* = 64)	Cardiac (*n* = 64)	To compare perioperative blood glucose and glycemic variability between added liraglutide and only-insulin infusion in DM patients undergoing cardiac surgery.
Song 2017 [54]	South Korea	Prospective cohort	Adults (*n* = 22)	Cardiac (*n* = 22)	To evaluate the accuracy and performance of the CGM system depending on different measurement sites in the OR and in the ICU.
Sugiyama 2018 [55]	Japan	Prospective cohort	Adults (*n* = 30)	Cardiac (*n* = 15), neuro (*n* = 15)	To evaluate the accuracy of a subcutaneous CGM system during different types of surgeries.
Sugiyama 2018 [56]	Japan	Case report	Child (*n* = 1)	Abdominal (*n* = 1)	To present a case in which real-time subcutaneous CGM, in combination with intermittent blood glucose measurement, was used for glycemic control during surgery for insulinoma resection.
Tripyla 2020 [57]	Switzerland	Prospective cohort	Adults (*n* = 20)	Abdominal (*n* = 20)	To assess the performance of the CGM system Dexcom G6 during elective abdominal surgery.
Vriesendorp 2005 [58]	The Netherlands	Prospective cohort	Adults (*n* = 8)	Abdominal (*n* = 8)	To examine whether CGM is feasible and reliable during and after major surgical procedures using two commercially available sensors.
Wasiq 2022 [59]	India	Prospective cohort	Neonates (*n* = 10)	Abdominal (*n* = 6), cardiac (*n* = 1), neuro (*n* = 2), urologic (*n* = 1)	To compare the blood glucose level by CGM with laboratory blood glucose testing in neonates during the perioperative period.

CGM, continuous glucose monitoring; POC, point-of-care; DM, diabetes mellitus; RCT, randomized controlled trial; OR, operating room; ICU, intensive care unit.

**Table 4:** Continuous glucose monitoring characteristics of the included studies (*n* = 22).

First Author Year	Manufacturer	Sensor Model	Location	Period CGM Values Collected
Aust 2014 [38]	Medtronic (Minneapolis, MN, USA)	CGMS system Gold	Subcutaneous	From 1 day before surgery to 72 h after surgery.
Blixt 2013 [39]	Eirus (Solna, Sweden)	Microdialysis system	Intravascular	OR to ward for a total of 20 h.
Carlsson 2023 [40]	Dexcom (San Diego, CA, USA)	G6	Subcutaneous	From 1 day before surgery to POD 8 or hospital discharge.
DiGiusto 2021 [41]	Dexcom (San Diego, CA, USA)	G6	Subcutaneous	OR to immediate postoperative.
Guensch 2021 [42]	Dexcom (San Diego, CA, USA)	G6	Subcutaneous	Intraoperative only.
Herzig 2023 [43]	Dexcom (San Diego, CA, USA)	G6	Subcutaneous	Intraoperative only.
Kalmovich 2012 [44]	Medtronic (Minneapolis, MN, USA)	CGMS system Gold	Subcutaneous	Intraoperative only.
Perez-Guzman 2021 [45]	Dexcom (San Diego, CA, USA)	G6	Subcutaneous	Perioperative.
Piper 2006 [46]	Medtronic (Minneapolis, MN, USA)	Guardian REAL-Time	Subcutaneous	From OR to a maximum of 72 h or until ICU discharge.
Polderman 2017 [47]	Edwards Lifescience (Irvine, CA, USA)	GlucoClear	Intravascular	From OR to PACU discharge.
Poljakova 2013 [48]	Medtronic (Minneapolis, MN, USA)	Guardian REAL-Time	Subcutaneous	From OR to 30 min after surgery.
Price 2023 [49]	Dexcom (San Diego, CA, USA)	G6	Subcutaneous	From OR to PACU discharge.
	Abbott (Abbott Park, IL, USA)	Freestyle Libre 2.0		
Saha 2018 [50]	Medtronic (Minneapolis, MN, USA)	Enlite	Subcutaneous	On the day of surgery.
Schierenbeck 2017 [51]	Eirus (Solna, Sweden)	Microdialysis system	Intravascular	From OR to POD 1.
	Abbott (Abbott Park, IL, USA)	Freestyle libre	Subcutaneous	From 1 day before surgery to POD 1.
Schierenbeck 2013 [52]	Eirus (Solna, Sweden)	Microdialysis system	Intravascular	From OR to 48 h after surgery or until catheter removal.
Sindhvananda 2023 [53]	Medtronic (Minneapolis, MN, USA)	Enlite	Subcutaneous	From 1 day before surgery to POD 3.
Song 2017 [54]	Medtronic (Minneapolis, MN, USA)	Guardian REAL-Time	Subcutaneous	From OR to 72 h after surgery or until ICU discharge.
Sugiyama 2018 [55]	Medtronic (Minneapolis, MN, USA)	Enlite	Subcutaneous	On the day of surgery.
Sugiyama 2018 [56]	Medtronic (Minneapolis, MN, USA)	Enlite	Subcutaneous	Intraoperative only.
Tripyla 2020 [57]	Dexcom (San Diego, CA, USA)	G6	Subcutaneous	From OR to 2 h after surgery.
Vriesendorp 2005 [58]	Medtronic (Minneapolis, MN, USA)	CGMS system Gold	Subcutaneous	Intraoperative only.
	A. Menarini Diagnostics (Florence, Italy)	GlucoDay	Subcutaneous	
Wasiq 2022 [59]	Abbott (Abbott Park, IL, USA)	Freestyle Libre	Subcutaneous	From 2 h before surgery to 72 h after surgery.

CGM, continuous glucose monitoring; OR, operating room; POD, postoperative day; ICU, intensive care unit; PACU, post-anesthetic care unit.

**Table 5:** Reported technical reliability outcomes of intraoperative continuous glucose monitoring (*n* = 18).

First Author Year	Sensor Model	Sensor Survival	Data Availability	Study Specific
Aust 2014 [38]	CGMS system Gold	10/10 (100%)	98.5%	NR
Carlsson 2023 [40]	G6	NR	96% (92, 98)	NR
DiGiusto 2021 [41]	G6	1/2 (50%)	NR	Failure to transmit data for a 30 min period shortly after induction in one patient.
Guensch 2021 [42]	G6	2/2 (100%)	NR	NR
Herzig 2023 [43]	G6	16/16 (100%)	90.1%	NR
Kalmovich 2012 [44]	CGMS system Gold	NR	NR	“Split curve” phenomenon: 10/32 (31%; defined as hypoglycemic values reported by CGM, but much higher values in actual blood glucose).
Piper 2006 [46]	Guardian REAL-Time	NR	NR	Device alarm: 10/20 (50%; due to use of electrocautery).
Polderman 2017 [47]	GlucoClear	NR	NR	Sensor failure: 9/37 (24.3%; defined as missing sensor data for > 50% of the intraoperative or postoperative period or when the difference from POC measurements on two consecutive time points was > 45 mg/dL)
Poljakova 2013 [48]	Guardian REAL-Time	17/17 (100%)	NR	NR
Price 2023 [49]	G6	64/76 (84.2%)	NR	NR
	Freestyle Libre 2.0			
Saha 2018 [50]	Enlite	1/1 (100%)	NR	NR
Schierenbeck 2017 [51]	Microdialysis system	NR	NR	Interruption: 22/24 (91.7%), data gap duration: 13 ± 19 min.
	Freestyle libre	NR	NR	Interruption: 1/24 (4.2%; due to excessive sweating causing sensor detachment).
Schierenbeck 2013 [52]	Microdialysis system	29/30 (96.7%)	NR	NR
Sindhvananda 2023 [53]	Enlite	60/64 (93.8%)	NR	NR
Song 2017 [54]	Guardian REAL-Time	Abdomen: 19/22 (86.4%)	Abdomen: 58.7%	NR
		Thigh: 22/22 (100%)	Thigh: 72.9%	
Sugiyama 2018 [55]	Enlite	1/1 (100%)	NR	NR
Tripyla 2020 [57]	G6	19/20 (95%)	98.6% (95.9, 100)	NR
Vriesendorp 2005 [58]	CGMS system Gold	7/8 (87.5%)	NR	Technical failure: 66% (defined as missing data).
	GlucoDay	8/16 (50%)		Technical failure: shoulder 10%, upper leg 63% (defined as missing data or broken fiber).

Values are expressed as mean ± SD, median (IQR), or proportion (%). NR, not reported; CGM, continuous glucose monitoring; POC, point-of-care.

**Table 6:** Reported accuracy outcomes of intraoperative continuous glucose monitoring (*n* = 18).

First Author Year	Sensor Model	Comparator Method	Matched Measurements	MARD (%)	Agreement (%)	Mean Bias (mg/dL)	Limits of Agreement (mg/dL)	Error Grid Analysis (%)	Correlation or Regression
Aust 2014 [38]	CGMS system Gold	Arterial BGA Capillary POC	Overall: 342	NR	NR	NR	NR	Clarke: 99.1	Pearson’s: 0.87 (95% CI: 0.844, 0.895)
			CPB: 59					Clarke: 100	Pearson’s: 0.76 (95% CI: 0.624, 0.851)
Blixt ^1^ 2013 [39]	Microdialysis system	Arterial CL	195	(1) 8.8 ± 8.4	NR	NR	(1) ± 42.1	(3) Clarke: 100	Pearson’s: 0.89 (*p* < 0.001)
				(2) 6.8 ± 9.3			(2) ± 34.9	(4) Clarke: 100	Pearson’s: 0.92 (*p* < 0.001)
DiGiusto ^2^ 2021 [41]	G6	Arterial BGA	NR	NR	NR	(3) 33.22 (4) 17.78	(3) 19.65 to 46.79 (4) 2.47 to 38.02	NR	R^2^: 0.9365 (*p* < 0.01) R^2^: 0.6057 (*p* < 0.01)
		Capillary POC				(3) 20.11 (4) 23.38	(3) 13.45 to 53.67 (4) 12.24 to 34.51		R^2^: 0.4752 (*p* = 0.0239) R^2^: 0.9095 (*p* < 0.01)
Guensch ^2^ 2021 [42]	G6	Venous BGA	16	(3) 4.3 ± 3.8 (4) 8.1 ± 5.6	NR	NR	NR	NR	NR
Herzig 2023 [43]	G6	Arterial BGA	Overall: 256	23.8	NR	NR	NR	Clarke: 86.3	NR
			ECC: 154	29.1					
			DHCA: 10	41.6					
Kalmovich 2012 [44]	CGMS system Gold	Venous BGA	NR	19.2	NR	NR	NR	NR	NR
Perez-Guzman 2021 [45]	G6	Arterial BGA, capillary POC	149	12.9	15/15: 69 20/20: 82 30/30: 94	NR	NR	Clarke: 98.6	NR
Piper 2006 [46]	Guardian REAL-Time	Arterial BGA	246	17.6	NR	NR	NR	Clarke: 98.8 Consensus: 99.6	Pearson’s: 0.787 (*p* < 0.001)
Polderman 2017 [47]	GlucoClear	Capillary POC	NR	7.8 [5.5, 10.4]	NR	−13.9	−64.3 to 36.6	NR	NR
Poljakova 2013 [48]	Guardian REAL-Time	Capillary POC	51	NR	NR	NR	NR	NR	Pearson’s: 0.866
Price 2023 [49]	G6 Freestyle Libre 2.0	Capillary POC	323	NR	NR	−18.27	−82.47 to 45.93	NR	Pearson’s: 0.731 (95% CI: 0.675, 0.778)
Schierenbeck 2017 [51]	Microdialysis system	Arterial BGA	514	6.5 ± 8.2	15/15: 90	0.9 ± 15.1	−27 to 29	Clarke: 100	NR
	Freestyle Libre		578	30.5 ± 12.4	15/15: 7	−43.4 ± 20	−82 to −4.5	Clarke: 99.1	
Schierenbeck 2013 [52]	Microdialysis system	Arterial BGA	607	5.6	20/15: 97.2	2.2	−10.4 to 14.8	Clarke: 100	NR
Song 2017 [54]	Guardian REAL-Time	Arterial BGA	Abdomen: 270	27.4 ± 20.1	NR	20.6	−143.8 to 185.0	Clarke: 89.0	Pearson’s: 0.45 (*p* < 0.001)
			Thigh: 331	29.7 ± 51.3		−7.8	−148.0 to 132.4	Clarke: 89.3	Pearson’s: 0.33 (*p* = 0.004)
Sugiyama 2018 [55]	Enlite	Arterial POC Capillary POC	Neuro: 144	NR	NR	−8.3	−37.1 to 20.6	Clarke: 100	NR
			Cardiac: 147			−23.5	−77.3 to 3.03	Clarke: 99.3	
Tripyla 2020 [57]	G6	Capillary POC	523	12.7 ± 5.4	15/15: 67.4 ± 24.5	9.0	−9.0 to 48.6	Clarke: 99.2 ± 2.6	NR
Vriesendorp ^3^ 2005 [58]	CGMS system Gold	Arterial BGA	NR	13	NR	NR	NR	Clarke: 100	NR
	GlucoDay			(5) 10 (6) 15				(5) Clarke: 99.1 (6) Clarke: 87.0	
Wasiq 2022 [59]	Freestyle Libre	Venous CL	40	NR	NR	23.8	−5.3 to 52.9	NR	Interclass: 0.953 (*p* < 0.001)
		Capillary POC				8.4	25 to 37.8		Interclass: 0.956 (*p* < 0.001)

Values are expressed as mean ± SD and median [IQR]. MARD, mean or median absolute relative difference; BGA, blood gas analysis; POC, point-of-care; CPB, cardiopulmonary bypass; NR, not reported; CL, central laboratory; ECC, extracorporeal circulation; DHCA, deep hypothermic circulatory arrest. ^1^ Two different methods of sensor calibration used in this article: 1 refers to calibration using the first plasma only, while 2 refers to recalibration to plasma glucose every 8 h. ^2^ Outcomes reported in two different patients: 3 refers to patient A, and 4 refers to patient B. ^3^ GlucoDay was placed at two different locations: 5 refers to the shoulder and 6 refers to the upper leg.

**Table 7:** Reported adverse device effects of intraoperative continuous glucose monitoring (*n* = 10).

First Author Year	Sensor Model	Predefined Definition	Incidence
Aust 2014 [38]	CGMS System Gold	No	0/10 (0%)
Blixt 2013 [39]	Microdialysis system	No	0/10 (0%)
Carlsson 2023 [40]	G6	No	0/70 (0%)
Herzig 2023 [43]	G6	No	0/0 (0%)
Piper 2006 [46]	Guardian REAL-Time	Adverse skin reaction, infection, or sensor dislodgment.	0/20 (0%)
Poljakova 2013 [48]	Guardian REAL-Time	No	0/17 (0%)
Schierenbeck 2013 [52]	Microdialysis system	No	0/30 (0%)
Song 2017 [54]	Guardian REAL-Time	Adverse skin reaction, infection, or bleeding.	0/44 (0%)
Wasiq 2022 [59]	Freestyle Libre	Local infection or thrombophlebitis.	0/10 (0%)
Tripyla 2020 [57]	G6	No	2/20 (10%) Due to self-limited bleeding after sensor insertion, mild pruritus, and skin irritation.

Values are expressed as proportion (%).

## Data Availability

No new data were created or analyzed in this study.

## Supplementary Materials

The following supporting information can be downloaded at: https://www.mdpi.com/article/10.3390/jcm13206169/s1, Table S1: Preferred Reporting Items for Systematic reviews and Meta-Analyses extension for Scoping Reviews (PRISMA-ScR) Checklist; Table S2: Complete search strategy. Reference [34] is cited in Supplementary Materials.
